# Supplementary material for: Epidemiology of Malnutrition among Children with Cerebral Palsy in Low- and Middle-Income Countries: Findings from the Global LMIC CP Register
Source: Nutrients. 2021 Oct 20;13(11):3676. doi: 10.3390/nu13113676 (PMC8619063; doi:10.3390/nu13113676)
Supplement: Supplementary file 1 [file nutrients-13-03676-s001.zip › nutrients-1401891-supplementary.pdf]

Supplementary files

Supplementary Table S1: Assessment of anthropometric measurements

**Anthropometric measurements**	**Methods**
Weight	Weight was measured in kilograms using digital weighing scale with a precision of 100 grams. For young children and children who could not stand independently, we documented the tared weight where, at first the weight of primary/accompanying caregiver of the child is measured (i.e. Wcaregiver), then combined weight of the child with their primary/accompanying caregiver is measured (i.e. Wcaregiver + Wchild). Weight of the child is calculated using following formula; Wchild= (Wcaregiver + Wchild) – (Wcaregiver). The standard procedure for weight measurements were followed (e.g. bare feet, minimum clothing). [1]
Length/Height:	For young children aged <2 years, we measured the length using a length board. For children aged ≥2 years we measured the height using height scale. If length/height could not be measured due to severe physical impairment, we measured the knee height using a measuring tape which was then converted to full height using the following formula: Height=2.69 x knee height + 24.2. [2] All measurements (i.e. length/height/knee height) were collected in centimeters following standard procedsure. [1]
Mid-Upper Arm Circumference (MUAC):	MUAC was measured in centimeters for children aged less than 61 months at assessment using a MUAC tape following standard procedure. [1]

References

1. WHO. Training course of Child Growth Assessment; WHO: China, 2008.
2. Stevenson, R.D. Use of segmental measures to estimate stature in children with cerebral palsy. Archives of pediatrics & adolescent medicine 1995, 149, 658–662

Supplementary Table S2: Nutritional status of children aged five years or less

Nutritional indicator	Bangladesh		Indonesia		Nepal		Ghana	
	Children with CP, n=1015, n (%)	National average [1] n (%)	Children with CP, n=37, n (%)	National average [2] n (%)	Children with CP, n=28, n (%)	National average [3] n (%)	Children with CP, n=226, n (%)	National average [4] n (%)
**Weight-for-age [5]**								
Normal	292 (29.5)	N/A	7 (18.9)	N/A	16 (61.5)	N/A	117 (56.3)	N/A
Underweight	698 (70.5)	21.9	30 (81.1)	N/A	10 (38.5)	27.0	91 (43.8)	11.0
**Height-for-age [5]**								
Normal	187 (19.3)	N/A	3 (8.3)	N/A	9 (39.1)	N/A	102 (50.0)	N/A
Stunted	782 (80.7)	30.8	33 (91.7)	N/A	14 (60.9)	35.8	102 (50.0)	18.8
**Weight-for-height [5]**								
Normal	587 (64.9)	N/A	18 (69.2)	N/A	19 (82.6)	N/A	106 (60.9)	N/A
Wasted	317 (35.1)	8.4	8 (30.8)	N/A	4 (17.4)	9.7	68 (39.1)	4.7
**MUAC-for-age [5]**								
Normal	723 (73.6)	N/A	27 (73.0)	N/A	18 (72.0)	N/A	107 (58.2)	N/A
Wasted	260 (26.4)	N/A	10 (27.0)	N/A	7 (28.0)	N/A	77 (41.8)	N/A

[1] Bangladesh Demographic and Health Survey 2018; [2] Not available (N/A); [3]Nepal Demographic and Health Survey 2016; [4]Ghana Demographic and Health Survey 2014; [5]missing data; [6]z score>+2SD were exclueded from the analysis

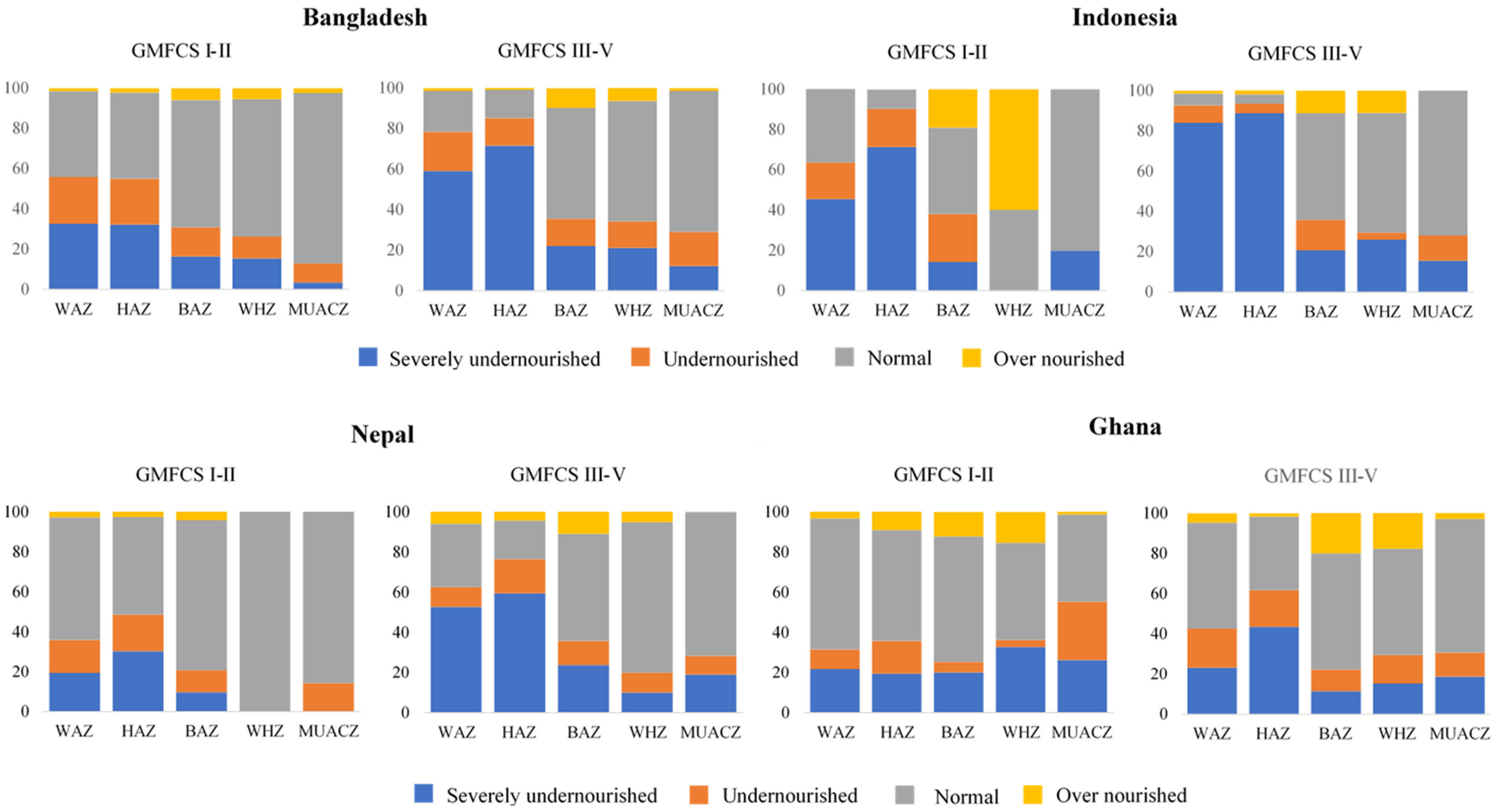

Supplementary Figure S1: Nutritional statua of children with CP according to their GMFCS level
